# 

**Published:** 1923-09

**Authors:** 


### PUBLIC HEALTH IN PARLIAMENT.

The following answers have been given for the Minister of Health.

### HOUSING.

Subsidy.—There is no authority for the statement that the grant by the State of £6 per house for 20 years will be dependent on the local authority making a contribution out of the local rates. Where the local authority with my approval themselves erect houses complying with the Bill the Government grant will in all cases be £6 for 20 years Where the local authority grant assistance to private enterprise, the Government grant will be £6 for 20 years if the total assistance so granted is equal to or greater than the equivalent of this annual payment. If the total assistance granted to private enterprise is less than the equivalent of £6 for 20 years, the Government contribution in accordance with the latter part of the proviso to Clause 1 of the Bill will be the equivalent of the total assistance actually granted.

### NATIONAL HEALTH INSURANCE.

Contributions,—The amounts of health insurance contributions were approximately as follows: In England and Wales in 1921, £22,130,000; in 1922, £22,500,000. In Scotland in 1921, £2,740,000; in 1922, £2,720,000. The shortage of contributions as compared with the amount assumed in the financial basis of the Act—about 48 contributions per insured person per annum—is estimated to be approximately 7 per cent. in England and Wales and 10 per cent. in Scotland.

### HOSPITAL ACCOMMODATION.

Poor Law Infirmaries.—I should be very glad to consider any proposals which may be put forward by Boards of Guardians and the authorities of the hospitals for the general use of surplus accommodation in poor law infirmaries.

### THE PUBLIC HEALTH.

Venereal Disease.—Chemists (Sale of Disinfectants).—I have consulted the Law Officers of the Crown as to the effect of sub-section 2 of section 2 of the Venereal Disease Act, 1917, and I am advised by them that there is nothing in the sub-section to prevent a chemist from selling disinfectants for the prevention of venereal disease where no written or printed instructions are affixed to or delivered with the disinfectant, or from giving verbal recommendations of medicinal preparations intended to be used or applied for the purpose.

Tuberculosis (Deaths).—The number of deaths from tuberculosis in England and Wales during the year 1921 was 42,678, and during the year 1922, 42,777.

Small-pox (Deaths).—The annual mortality per million living from small-pox for the periods 1861–70, 1871–80, 1881–90, 1891–1900, 1901–10 and 1911–20 for the usual age-groups, for ages under 15, viz.: 0–5, 5–10 and 10–15, is shown in the table below:—

| | 0–5. | 5–10. | 10–15. |
|---|---|---|---|
| 1861–70 | 638 | 143 | 56 |
| 1871–80 | 518 | 285 | 138 |
| 1881–90 | 80 | 33 | 26 |
| 1891–1900 | 29 | 10 | 3 |
| 1901–10 | 22 | 7 | 6 |
| 1911–20 | 0.57 | 0.32 | 0.11 |

### RECENT APPOINTMENTS.

### Medical Officers, etc.

Barkas, M. R., M.B., B.Sc., M.Sc.; Assistant M.O., Maudsley Hospital.

Bennett, James, M.R.C.S., L.R.C.P., D.P.H., R.C.P.S.; M.O.H., Warrington.

Berry, Ronald B., M.D., D.P.H., Assistant M.O.H., Lancashire County Council; M.O.H. and School M.O., Gloucester, £1,000.

Bird, G. W. Harvey, M.R.C.S., L.R.C.P., M.B., B.C.; M.O.H. and School Medical Officer, Bridgwater.

Cardell, J. D. M., M.R.C.S., L.R.C.P.; Senior Resident Anæsthetist, St. Thomas's Hospital.

Clark, James Alexander Macdonald, M.B., Ch.B., M.,.D D.P.H., R.C.P.S. Edin., R.F.P.S., Glasgow, Tuberculosis Officer, Borough of Walsall; M.O.H., Borough of Walsall (£900 and qualified assistant).

Edwards, Thomas Peters, M.B., B.S., M.D.(Lond.), D.P.H. (Camb.), Senior Assistant M.O. Denbighshire; M.O., Wrexham District. (£700 and travelling expenses, rising by annual increments of £25 to £800.)

Fry, G. (Mrs.), M.B., B.S., D.P.H.; M.O., Welwyn Garden City Infant Welfare Centre.

Hill, H. Gardiner, M.B.E., M.R.C.S., L.R.C.P.; Senior Casualty Officer (Medical), St. Thomas's Hospital.

Patrick, C. V., M.R.C.S., L.R.C.P.; Senior Casualty Officer (Surgical), St. Thomas's Hospital.

Potter, A. F., M.R.C.S., L.R.C.P.; Resident M.O., St. Thomas's Home.

Ranson, J. S., M.R.C.S., L.R.C.P., Assistant M.O.H., Ipswich; M.O.H., Halstead and Belchamp United Districts, Essex.

Reid, Margaret A., M.B., Ch.B. (Aberdeen), Assistant M.O., St. Helens; Assistant Welfare M.O. County of Durham (Aberdeen University).

Saunders, J. C., M.B., D.P.H., Assistant Tuberculosis Officer, Borough of Warrington; Assistant M.O.H., Croydon.

Schuster, Norah, M.B., Ch.B.; Pathologist, the Infants' Hospital, Vincent Square, Westminster.

Southam, Arthur Hughes, M.B., B.Ch., Oxford, M.R.C.S. Eng., L.R.C.P., London; Hon. Surgeon, Manchester Jewish Hospital.

Stevenson, John, Medical Superintendent, Prestwood Sanatorium; £800, with house, lighting and fuel.

Walker, Mary Carswell, M.B., Ch.B., D.P.H., R.C.P.S., R.F.P.S., Assistant Medical Officer, Perthshire.

Zimmerman, Richard I., House Physician, St. Bartholomew's Hospital, Rochester.

### Public Health and Hospital Appointments.

Attenborough, S. L., Matron, Walford Infants' Home (King's College Hospital).

Austin, Kathleen, C.M.B.; County Health Superintendent, Gloucestershire County Council.

Collier, D., C.M.B., Health Visitor, Folkestone (Farnham Infirmary).

Cullen, F. A., Assist. Sanitary Inspector, Birmingham; Sanitary Inspector, Bognor Rural District Council.

Dare, Edith G.; Matron, Queen Charlotte's Hospital (Newport and County Hospital, Monmouthshire).

Dinsley, Kathleen, Assistant Supt. Health Visitor, County Bristol; Supt. Health Visitor, County of Surrey (Bristol General Hospital).

Firth, J., Matron Aylesbury Isolation Hospital (Greenock General Infirmary).

Fisher, J. S.; Sanitary Inspector, St. Columb Rural District Council.

Forsyth, Amy M., Ward Sister and Assistant Night Superintendent, Edinburgh Royal Infirmary; Matron, Edinburgh Maternity Hospital (Edinburgh Royal Infirmary).

Garner, J. H., B.Sc., M.I.Ch.E., Manager and Chemist, Huddersfield Sewage Disposal Works; Chief Chemist, West Riding Rivers Board. (£600.)

Hart, J. W., Surveyor, Golborne Urban District Council; Sanitary Inspector, Golborne Urban District Council.

MacManus, Emily E. P., C.M.B.; Matron, Bristol Royal Infirmary.

Maslin, G. C., Sanitary Inspector, Darmley, Glos.

Nichols, A. J., District Sanitary Inspector, Wood Green; Sanitary Inspector, Luton.

Owen, B. J., of Manselton; Sanitary Inspector, Swansea Rural District Council.

Pope, Mabel, A.R.R.C., Assistant Matron, Q.V. Jubilee Convalescent Home, Bristol; Matron thereof (Bristol General Hospital).

Richards, Elizabeth A., A.R.R.C.; Matron, Benenden National Hospital (Middlesex Hospital).

Thomas, Claudia, Health Visitor, Caerphilly.

Thomas, Florence V., Health Visitor, Stockton-on-Tees (Eccleshall Infirmary, Sheffield).

Weeks, H. J.; Sanitary Inspector, Stratford-on-Avon.

Whitworth, C., Sanitary Inspector, Spilsby Rural District Council; Sanitary Inspector, Wayland Rural District Council.

